# Hypothalamic Lipoma: Outcome of an Intracranial Developmental Lesion

**DOI:** 10.1155/2022/7216090

**Published:** 2022-01-15

**Authors:** Ali Alkhaibary, Noura Alsubaie, Ahoud Alharbi, Noor Alghanim, Laila Baydhi, Sami Khairy, Fahd Musawnaq, Abdulaziz Alarifi, Mohammed Alwohaibi, Ahmed Aloraidi, Makki Almuntashri, Ahmed Alkhani

**Affiliations:** ^1^College of Medicine, King Saud bin Abdulaziz University for Health Sciences, Riyadh, Saudi Arabia; ^2^King Abdullah International Medical Research Center, Riyadh, Saudi Arabia; ^3^Division of Neurosurgery, Department of Surgery, King Abdulaziz Medical City, Ministry of National Guard-Health Affairs, Riyadh, Saudi Arabia; ^4^College of Medicine, Alfaisal University, Riyadh, Saudi Arabia; ^5^College of Medicine, Jazan University, Jazan, Saudi Arabia; ^6^Medical Imaging Department, King Abdulaziz Medical City, Ministry of National Guard-Health Affairs, Riyadh, Saudi Arabia

## Abstract

**Background:**

Hypothalamic lipomas are benign developmental lesions that tend to be discovered incidentally. This article describes the radiological features, outcome, and the postulated theories behind hypothalamic lipomas development.

**Methods:**

The electronic archive of neurosurgery was retrospectively reviewed. All patients with a neuroradiological diagnosis of hypothalamic lipoma, between 2005 and 2020, were included.

**Results:**

Out of 246 patients with intracranial lipomas, a total of six patients with hypothalamic lipomas have been identified. On computed tomography images, one of the hypothalamic lipomas demonstrated calcification. On magnetic resonance imaging, peripheral enhancement after contrast administration was noted in one of the lesions. Considering the benign nature of the lesions, neurosurgical intervention was not indicated.

**Conclusion:**

The majority of patients with hypothalamic lipomas are asymptomatic and undergo brain imaging for other indications. Although uncommon, such developmental lesions can be identified in the general population, especially with the advancement of neuroimaging techniques.

## 1. Introduction

Intracranial lipomas are uncommon benign lesions comprising less than 1% of intracranial tumors [1–3]. They are commonly located in the midline and associated with midline brain deformities [4]. Intracranial lipomas have been proposed to be secondary to proliferation of normal adipose cells present in the leptomeninx, deposition of fats as products of neuronal tissue, or persistence of displaced dermal anlage that later forms the lipoma within the neural tube [3]. Another theory proposed that intracranial lipomas are due to the persistence of mesenchymal tissue that gives rise to the meninges “meninx primitiva” which is thought to be a derivative of the neural crest [3]. Intracranial lipomas are usually asymptomatic and do not necessitate treatment [3]. Surgical intervention is preserved for patients with neurological deficits [5]

The incidental finding of intracranial lipomas is uncommon. Considering their poor discussion in the literature, the present article outlines the radiological findings and outcome of hypothalamic lipoma.

## 2. Methods

The radiological and departmental database of neurosurgical patients were reviewed from the period of 2005-2020. A retrospective review of all patients with hypothalamic lipoma was conducted. The available imaging studies were retrieved for radiological characterization of the lesions. Each patient is described in terms of radiological findings and outcomes.

## 3. Ethical Considerations

Patients' identities were concealed. All radiological images were anonymized. The study was noninterventional and retrospective in nature.

## 4. Results

Of 246 patients with intracranial lipomas, a total of six patients with hypothalamic lipomas of variable imaging appearances have been identified from the period of 2005-2020. The youngest patient was 16 years old (age range = 16 − 60 years) at the time of diagnosis. The mean age of all patients was 33.5 ± 13.56 years. There was no gender predilection (males; *N* = 3, females; *N* = 3). All patients had no signs or symptoms of endocrinopathy. Calcification and peripheral enhancement after contrast demonstration were noted in two patients with hypothalamic lipoma.  Table 1 outlines the characteristics of patients with hypothalamic lipoma in the present case series.

### 4.1. Patient 1

**Figure 1:**
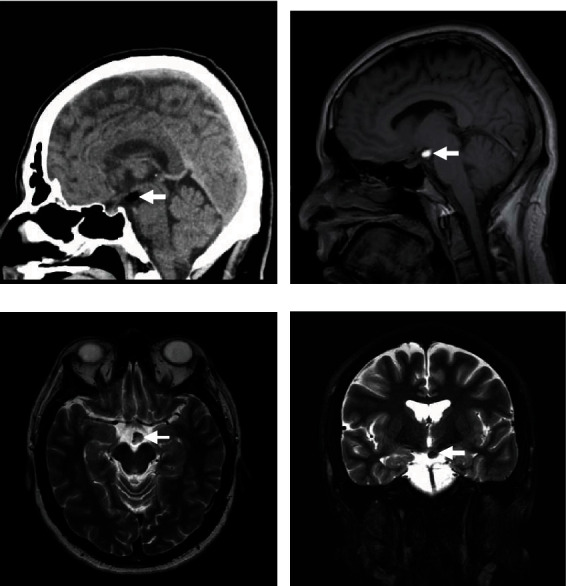
(a) Sagittal brain CT without contrast. (b) Sagittal T1-weighted MRI. (c) Axial T2-weighted MRI. (d) Coronal T2-weighted fat-saturated MRI. (a)–(d) The images demonstrate a homogeneously oval-shaped, fat-containing suprasellar hypothalamic lesion measuring 11 × 7 × 7 mm in the anterior-posterior, transverse, and craniocaudal dimensions, respectively, on the left side. The lesion is hyperintense in T1-weighted images, intermediate to hyperintense in T2-weighted images, and suppressed on fat-saturated images. No internal septation, nodularity, or calcifications are noted.

A 27-year-old male, known to have asthma, dextrocardia, hypertension, and hyperthyroidism, presented to the neurosurgery outpatient clinic due to intermittent episodes of mild-intensity headache. The headache was mainly triggered by exposure to allergens during certain seasonal times of the year. The patient reported no history of vomiting, seizures, loss of consciousness, dizziness, facial pain/swelling, recent ear infection, postnasal drip, gross obesity, history of precocious puberty, blurry vision, or any other associated symptoms suggestive of high intracranial pressure. In the clinic, the patient's physical examination revealed that he was alert and oriented to person, place, and time with a Glasgow Coma Scale (GCS) of 15/15. Pupils were 3 mm bilaterally reactive to light with no gaze preference. The remainder of the physical examination was grossly intact. Computed tomography (CT) scan revealed a homogeneously fat-containing lesion in the hypothalamus (Figure 1(a)). Magnetic resonance imaging (MRI) without and with contrast showed a stable, small (10 mm) hypothalamic lipoma with no pathological enhancement (Figures 1(b)–1(d)).

### 4.2. Patient 2

**Figure 2:**
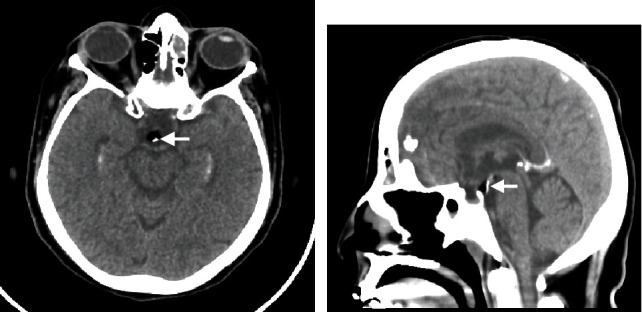
(a) Axial brain CT. (b) Coronal brain CT. (a) and (b) The images demonstrate a small fat-density lesion with punctate calcification, representing a hypothalamic lipoma.

A 60-year-old female, known to have diabetes, coronary artery disease, hypothyroidism, dyslipidemia, and cholelithiasis, was referred from the emergency department to ophthalmology clinic due to progressive decrease in visual acuity. Physical examination was grossly intact. A nonenhanced brain computed tomography revealed a hypodense lesion with a predominant fatty component and a focus of calcification (Figure 2). The patient was referred to neurosurgery clinic for further assessment. However, she did not show up to her neurosurgery or ophthalmology appointments since her initial ophthalmological assessment.

### 4.3. Patient 3

**Figure 3:**
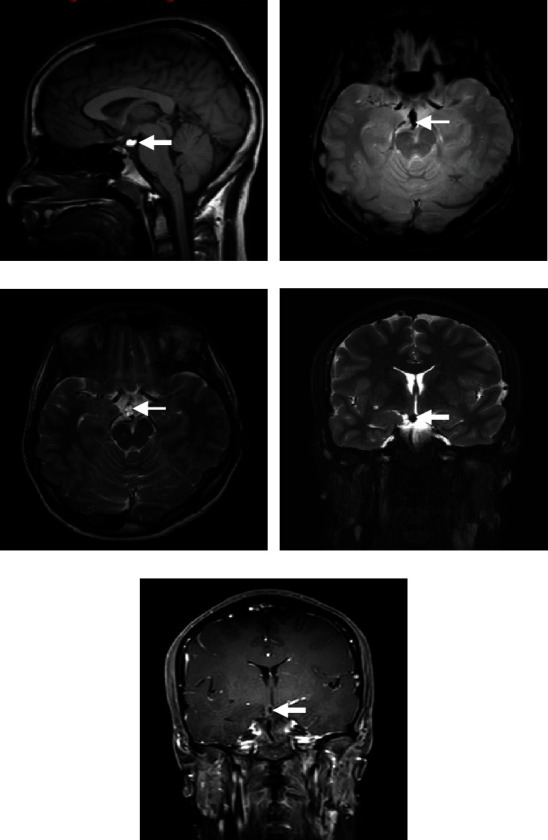
(a) Sagittal T1-weighted MRI. (b) Axial T2-gradient MRI. (c) Axial T1-weighted MRI. (d) Coronal T2-weighted MRI. (e) Coronal T1-weighted MRI with contrast. (a)–(e) The images are demonstrating a well-defined hypothalamic lesion following fat signal on all sequences. Faint peripheral enhancement is noted after contrast administration.

A 16-year-old male, not known to have any medical illness, presented to the emergency department due to episodic vasovagal syncope after the sight of blood. There was no history of headache, dizziness, abnormal movements, or seizures. Neurological examination was intact. A brain MRI revealed a hypothalamic lipoma (Figure 3).

### 4.4. Patient 4

**Figure 4:**
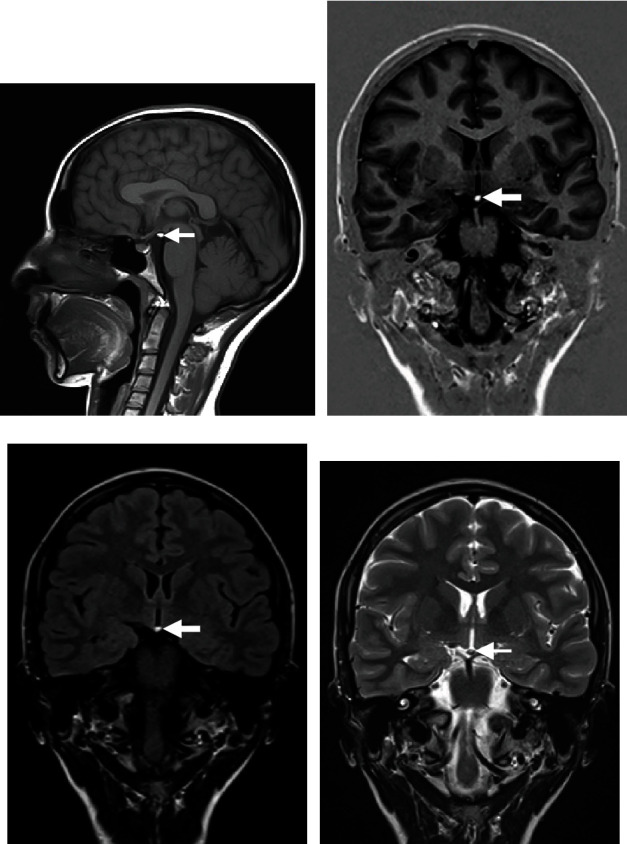
(a) Sagittal T1-weighted MRI. (b) Coronal T1-weighted gradient MRI (FSPGR). (c) Coronal FLAIR T2-weighted MRI. (d) Coronal T2-weighted image. (a)–(d) A small hypothalamic lipoma is noted, following fat signal on all sequences.

A 26-year-old female, known to have epilepsy diagnosed at the age of 18 years, presented with bilateral myoclonic abnormal jerky movements involving the upper limbs for five minutes. Afterward, the abnormal movements secondarily generalized to involve all limbs for a total of ten minutes. Physical examination, after recovery from the seizure event, was grossly intact. A brain MRI, utilizing epilepsy protocol, revealed a small hypothalamic lipoma (Figure 4).

### 4.5. Patient 5

**Figure 5:**
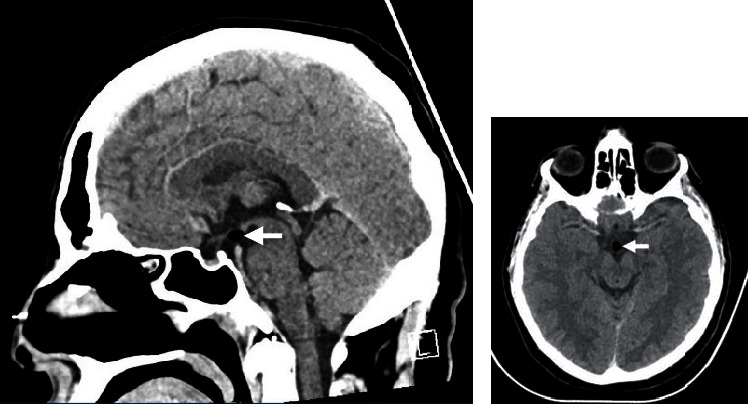
(a) Sagittal brain CT. (b) Axial brain CT. (a) and (b) The images demonstrate a small hypodense lesion, measuring 5 × 7 mm, consistent with hypothalamic lipoma.

A 36-year-old male, not known to have any medical illness, was complaining of intermittent band-like headache. The headache was associated with nausea and dizziness. Physical examination was grossly intact. Brain CT scan revealed an incidental hypothalamic lipoma (Figure 5).

### 4.6. Patient 6

**Figure 6:**
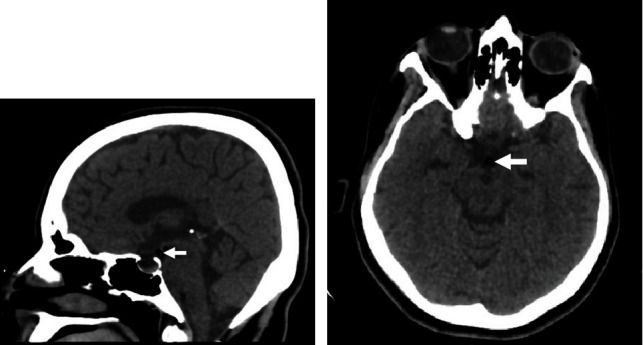
(a) Sagittal brain CT. (b) Axial brain CT. (a) and (b) A hypothalamic lipoma is noted measuring 4 × 4 × 4 mm in maximal dimensions.

A 36-year-old female, not known to have any medical illness, presented to the emergency department complaining of moderate-intensity headache. Physical examination did not reveal any deficits. Brain CT demonstrated a hypothalamic lipoma (Figure 6).

## 5. Discussion

Hypothalamic lipomas in the adult and pediatric populations are rare. A review of the literature revealed few reported cases. We hereby report a retrospective review comprising six patients with incidental hypothalamic lipomas who were identified at our medical institute.

As far as the embryologic concepts are concerned, hypothalamic lipomas are neither hamartomas nor neoplasms [6]. Rather, they develop due to congenital malformations [6]. Lipomas can be secondarily ossified, leading to the development of osteolipomas [6]. These lesions commonly tend to consist of mature adipose tissues and bony elements [6].

Hypothalamic lipomas are typically asymptomatic lesions, that is, discovered incidentally [7]. However, large-sized lesions may compress adjacent structures producing focal neurological deficits [7]. Additionally, hypothalamic lipomas have been well documented to contribute to hypothalamic dysfunction, including precocious puberty, hypothermia, and obesity [8–10]. In the absence of endocrinological, nutritional, or genetic dysfunction, hypothalamic lipoma can be the culprit of unexplained obesity [10]. Puget et al. highlighted the importance of performing magnetic resonance imaging in patients with unexplained obesity to identify the presence of hypothalamic lipoma [10].

On neuroimaging studies, hypothalamic lipomas depict signal intensity similar to those of subcutaneous fat tissues on magnetic resonance imaging [7]. The lesions commonly demonstrate homogenous intensity without enhancement after contrast administration [7]. In rare cases, they may demonstrate a focus of superficial calcification that is best detected on computed tomography images, as shown in Figure 2 [7].

In the present case series, the diagnosis of hypothalamic lipoma was rendered after brain CT and/or MRI in all patients. The lesions were fat-suppressed in fat-saturated T2-weighted images. One of the lesions demonstrated peripheral enhancement following contrast administration. Follow-up images, in all patients, showed stable hypothalamic lipomas. Considering the benign nature of the lesions, neurosurgical intervention was not indicated. All patients had excellent outcome. Except for one patient, they are currently followed-up at the neurosurgery clinic.

## 6. Conclusion

The majority of patients with hypothalamic lipomas are asymptomatic and undergo brain imaging for other indications. The characteristic imaging features of hypothalamic lipomas on brain computed tomography and magnetic resonance imaging help narrow down the differential diagnoses in patients with hypothalamic lesions. Patients with hypothalamic lipoma are commonly managed conservatively with regular clinical and radiological follow-up. Although uncommon, such lesions can be identified in the general population, especially with the advancement of neuroimaging techniques.

## Figures and Tables

**Table 1:** Characteristics of patients with hypothalamic lipoma identified after radiological imaging.

No.	Age^a^/sex	Presentation	Neurological examination	Radiological findings			Management	Follow-up duration
				Size^b^	Calcification	Enhancement		
1	27/M	Headache	Intact	11 × 7 × 7 mm	**—**	**—**	Conservative	57 months
2	60/F	Decreased visual acuity	Intact	8 × 7 × 5 mm	**+**	**—**	Not available	Lost to follow-up
3	16/M	Syncope	Intact	9 × 4 × 5 mm	**—**	**+**	Conservative	9 months
4	26/F	Epilepsy	Intact	4 × 3 × 2 mm	**—**	**—**	Conservative	30 months
5	36/M	Headache	Intact	7 × 8 × 5 mm	**—**	**—**	Conservative	4 months
6	36/F	Headache	Intact	4 × 4 × 4 mm	**—**	**—**	Conservative	4 months

^**a**^Age at initial identification of the hypothalamic lipoma. ^b^Size of the lesion measured in millimeters in anterior-posterior, transverse, and craniocaudal dimensions. M: male; F: female; (+): present; (-): absent.

## Data Availability

Data is available upon request.

## Abbreviations

CT: Computed tomography
GCS: Glasgow coma scale
MRI: Magnetic resonance imaging.

## References

[1] Jabot G., Stoquart-Elsankari S., Saliou G., Toussaint P., Deramond H., Lehmann P. (2009). Intracranial lipomas: clinical appearances on neuroimaging and clinical significance. *Journal of Neurology*.

[2] Loddenkemper T., Morris H. H., Diehl B., Lachhwani D. K. (2006). Intracranial lipomas and epilepsy. *Journal of Neurology*.

[3] Truwit C. L., Barkovich A. J. (1990). Pathogenesis of intracranial lipoma: an MR study in 42 patients. *AJR. American Journal of Roentgenology*.

[4] Donati F., Vassella F., Kaiser G., Blumberg A. (1992). Intracranial lipomas. *Neuropediatrics*.

[5] Eghwrudjakpor P. O., Kurisaka M., Fukuoka M., Mori K. (1991). Intracranial lipomas. *Acta Neurochirurgica*.

[6] Wittig H., Kasper U., Warich-Kirches M., Dietzmann K., Roessner A. (1997). Hypothalamic osteolipoma. *A case report. Gen Diagnostic Pathology*.

[7] Bonneville F., Cattin F., Marsot-Dupuch K., Dormant D., Bonneville J. F., Chiras J. (2006). T1 signal hyperintensity in the sellar region: spectrum of findings. *Radiographics*.

[8] Bognár L., Bálint K., Bárdóczy Z. (2002). Symptomatic osteolipoma of the tuber cinereum: case report. *Journal of Neurosurgery*.

[9] Di Pietro P., Debbia C., Fondelli M. P. (2004). Pediatric hypothalamic lipoma with hypothermia--case report. *Brain & Development*.

[10] Puget S., Garnett M. R., Leclercq D. (2009). Hypothalamic lipoma associated with severe obesity: report of 2 cases. *Journal of Neurosurgery. Pediatrics*.

